# Methods to Study Mitochondrial Metabolism and Homeostasis in Fission Yeast

**DOI:** 10.1002/yea.70030

**Published:** 2026-06-08

**Authors:** Ferran Gómez‐Armengol, Elena Hidalgo, Montserrat Vega

**Affiliations:** ^1^ Oxidative Stress and Cell Cycle Group Universitat Pompeu Fabra Barcelona Spain

## Abstract

- *Schizosaccharomyces pombe* offers a powerful model for mitochondrial studies
- In vivo and in vitro methods to study mitochondrial function and homeostasis
- Assays to study mitochondrial metabolism, bioenergetics, and redox balance
- Description of biosensors for in vivo measurement of ATP, H_2_O_2_, NAD(P)H
- Tools to analyze morphology and respiratory supercomplexes.

1

Mitochondria are indispensable organelles in eukaryotic cells under aerobic growth conditions, where they serve as major hubs for energy production. Through oxidative phosphorylation, mitochondria generate ATP, supporting cellular growth and proliferation. In addition to their central metabolic role, mitochondria participate in a wide range of essential cellular processes, including iron–sulfur cluster biogenesis, synthesis of the heme group, redox homeostasis, and the generation of metabolic intermediates required for amino acid, lipid and nucleotide biosynthesis. Mitochondria are therefore not only bioenergetic organelles but also key integrators of cellular metabolism and signaling.

Given their multifaceted functions, mitochondrial activity must be tightly regulated and coordinated with cellular demands. Perturbations in mitochondrial metabolism or homeostasis can have profound consequences for cell physiology, impacting growth, stress responses, aging, and survival. As a result, understanding how mitochondrial function is regulated under different environmental and nutritional conditions remains a central question in cell biology.

*Schizosaccharomyces pombe* is a complementary model system to budding yeast for investigating mitochondrial metabolism and homeostasis. It has several advantages, such as the excellent knowledge database (Pombase) (Rutherford et al. 2024) and the existence of a commercial deletion collection (Bioneer) (Kim et al. 2010). Furthermore, it is closer to human mitochondrial physiology than *Saccharomyces cerevisiae* (for a review, see [Dinh and Bonnefoy 2024]). We will here briefly summarize some in vivo and in vitro approaches which have been used in fission yeast and other eukaryotes to study mitochondrial metabolism (Section 1) and mitochondrial homeostasis (Section 2). At the beginning of each section, we will highlight the specific features that define *S. pombe* as an important alternative model to study mitochondria.

## 2 Monitoring Mitochondrial Metabolism

Glucose can follow three principal metabolic fates: it can be oxidized to pyruvate through glycolysis and subsequently reduced via fermentative pathways, leading to ethanol production; alternatively, glycolysis‐derived pyruvate can be transported into mitochondria, where it is oxidized to acetyl‐CoA and further metabolized through the tricarboxylic acid (TCA) cycle and oxidative phosphorylation; or glucose can be diverted from glycolysis at the level of glucose‐6‐phosphate into the pentose phosphate pathway. During fermentation, the NADH generated during glycolysis is reoxidized, yielding two molecules of ATP per molecule of glucose. In contrast, the TCA cycle provides intermediates for amino acid, lipid, and nucleotide biosynthesis, as well as reducing equivalents in the form of NADH and FADH_2_ that feed the electron transport chain (ETC). The ETC uses these reducing equivalents to generate a proton gradient across the inner mitochondrial membrane, which is subsequently harnessed by ATP synthase to produce ATP, producing up to 17–18 ATP molecules per glucose molecule (Verduyn et al. 1991). In most yeasts, there is no respiratory complex I; they only contain NADH dehydrogenase enzymes that lack proton‐pumping activity. The routing of pyruvate toward either fermentation or mitochondrial respiration represents a key metabolic decision that depends on environmental conditions. In many yeasts, such as *S. pombe* and *S. cerevisiae*, aerobic conditions combined with high glucose availability favor fermentative metabolism, while mitochondrial respiration is repressed, a phenomenon known as the Crabtree effect (De Deken 1966). Under nutrient‐limited conditions, however, this balance between fermentation and mitochondrial respiration shifts toward respiration in order to maximize energy yield from glucose (Gomes and Scorrano 2013; Takeda et al. 2015).

The importance of mitochondrial respiration varies among yeast species. Petite‐positive yeasts, such as *S. cerevisiae*, can proliferate in the absence of oxidative phosphorylation or even without mitochondrial DNA (Whittaker 1979). Budding yeast has evolved adaptations that allow it to bypass the requirement for mitochondrial respiration while maintaining essential biosynthetic functions, primarily through two mechanisms: the operation of a branched TCA cycle that supplies key metabolic intermediates (Camarasa et al. 2003), and the activation of the glyoxylate cycle (Liu and Butow 1999), which enables the net synthesis of TCA intermediates from C_2_ units. In contrast, petite‐negative yeast, such as *S. pombe*, is more similar to mammalian cells and cannot survive without mitochondrial respiration (Bulder 1964). Even under fermentative conditions, respiration in fission yeast is never fully repressed, allowing a partially functional TCA cycle and oxidative phosphorylation to sustain the production of amino acids essential for cell proliferation (Malecki et al. 2016; Malecki et al. 2020). Given its dependence on mitochondrial respiration, *S. pombe* provides a particularly powerful model system to investigate mitochondrial metabolism, dynamics, and quality control. Accordingly, this review focuses on experimental approaches applied in fission yeast to study mitochondrial metabolism and homeostasis.

### 2.1 In Vivo Experimental Approaches to Monitor Metabolism

**Figure 1:**
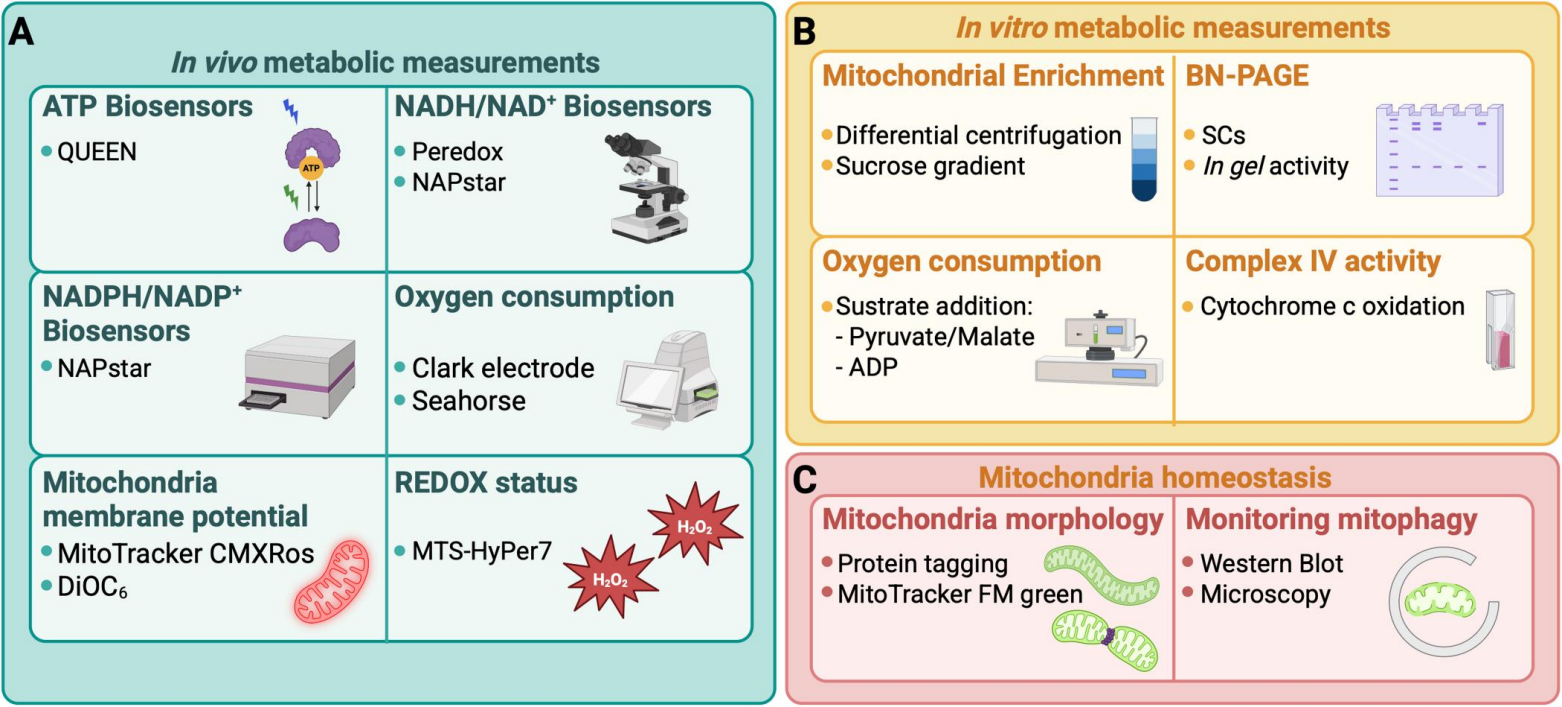
Schematic representation of the principal methodologies used in *S. pombe* to assess mitochondrial metabolism in vivo (A), in vitro (B), and mitochondrial homeostasis (C).

Within this section, we will highlight a variety of tools which have been developed to investigate different aspects of metabolism in yeast, including measurements of energy state (Section 1.1.1; ATP and NAD(P)H/NAD(P)+ biosensors), oxygen consumption (Section 1.1.2), mitochondrial membrane potential (Section 1.1.3), and redox state (Section 1.1.4). A schematic view of these approaches is included in Figure 1A.

#### 2.1.1 Energy State

Understanding how glucose is channeled toward fermentation or mitochondrial respiration, and how these pathways respond to specific environmental or nutritional conditions, requires experimental approaches that allow the precise monitoring of cellular metabolic states.

##### 2.1.1.1 ATP Biosensors

QUEEN is a genetically encoded fluorescent ATP sensor. QUEEN is based on a circularly permuted enhanced green fluorescent protein (cpEGFP) inserted into the bacterial FoF_1_‐ATP synthase ε subunit. It is a ratiometric biosensor that allows the absolute quantification of ATP levels in living cells, providing insights into how glucose is metabolized under different genetic backgrounds or nutritional conditions (Yaginuma et al. 2014). QUEEN has been successfully expressed in *S. pombe* from an integrative plasmid under the control of the strong constitutive *tif51* promoter (Takaine et al. 2019).

QUEEN enables not only the quantification of basal ATP levels but also the identification of the source of ATP production, distinguishing between glycolytic and mitochondrial contributions. This is achieved through the application of specific metabolic inhibitors: 2‐deoxyglucose, a non‐phosphorylatable glucose analog, blocks glycolysis and thereby total ATP production, revealing cellular ATP reserves; antimycin A, an inhibitor of mitochondrial complex III, selectively inhibits mitochondrial respiration.

Fluorescence is measured at two excitation wavelengths (410 nm and 480 nm), and the resulting ratio provides a quantitative assessment of ATP levels and their origin. These measurements can be performed at the single‐cell level using fluorescence microscopy or in bulk with plate readers. While QUEEN has been successfully expressed in the cytosol of fission yeast, its usefulness in mitochondria has not yet been demonstrated (Takaine et al. 2019).

##### 2.1.1.2 Redox State: NAD(P)H Biosensors

The cellular redox state is a key indicator of metabolic activity and pathway utilization. Genetically encoded fluorescent biosensors such as Peredox and NAPstar have been developed to monitor intracellular NAD(P)H dynamics in living cells. Peredox and derivatives are genetically encoded sensors that report changes in the NADH/NAD^+^ ratio through fluorescence intensity changes, providing information on the balance between glycolytic and respiratory metabolism. In contrast, the NAPstar biosensors are designed to monitor NADPH levels, enabling the analysis of anabolic metabolism and pathways such as the pentose phosphate pathway (Scherschel et al. 2024). These biosensors have not been expressed in *S. pombe* yet, and whether these families of biosensors maintain their functionality in the mitochondrial matrix has not been demonstrated. Of note, these biosensor‐based methodologies are the only ones in this review which have not yet been adapted to fission yeast.

#### 2.1.2 Oxygen Consumption—Clark Electrode and Seahorse Analyzers

In yeast, oxygen consumption rates reflect the activity of the mitochondrial ETC and provide a direct readout of respiratory metabolism under different nutritional conditions or genetic backgrounds. Respiratory activity can be measured using oxygen electrodes, such as Clark‐type electrodes (e.g., Oxygraph systems). The Oroboros O2k platform, also based on Clark‐type technology, enables high‐resolution respirometry and can be coupled to additional measurements such as ATP potential or ROS production. From these measurements, oxygen consumption rates can be calculated and normalized to cell number or biomass, allowing quantitative comparisons of respiratory activity (Divakaruni and Jastroch 2022; Panessa et al. 2023; Zuin et al. 2008). Alternatively, respirometry platforms such as Seahorse analyzers enable high‐throughput measurements of oxygen consumption in living cells by detecting changes in oxygen concentration in microchambers, providing real‐time assessments of basal respiration and respiratory responses to metabolic perturbations (Kumar et al. 2024; Berraquero et al. 2025). In both approaches, pharmacological agents can be used to interrogate mitochondrial respiratory function. Antimycin A, which inhibits mitochondrial complex III, blocks electron flow through the respiratory chain and thereby abolishes oxygen consumption. In contrast, the protonophore FCCP acts as an uncoupler by dissipating the proton gradient across the inner mitochondrial membrane (Groger et al. 2023). This uncoupling relieves the backpressure exerted by the proton motive force on the ETC, allowing maximal electron flux and oxygen consumption independently of ATP synthesis. Therefore, FCCP treatment, reveals the maximal respiratory capacity of the cells.

#### 2.1.3 Mitochondrial Membrane Potential

The mitochondrial membrane potential (ΔΨm) is a central parameter of mitochondrial function, reflecting the activity of the ETC. In yeast, ΔΨm can be assessed using fluorescent, membrane potential–sensitive dyes that accumulate within mitochondria in a potential‐dependent manner (Neikirk et al. 2023). Two widely used dyes in fission yeast are MitoTracker CMXRos and 3,3′‐dihexyloxacarbocyanine iodide (DiOC_6_).

MitoTracker Red CMXRos is a cell‐permeable x‐rosamine derivative containing a thiol‐reactive chloromethyl functional group that labels mitochondria. Its initial mitochondrial accumulation depends on the mitochondrial membrane potential, after which the dye reacts with thiol groups on mitochondrial proteins, resulting in covalent retention of the fluorescent signal. The mitochondrial membrane potential is tightly linked to respiratory activity: increased mitochondrial respiration generally leads to higher membrane polarization and enhanced dye accumulation, whereas reduced respiratory activity results in decreased staining (Groger et al. 2023).

DiOC_6_ is a lipophilic cationic carbocyanine dye that passively distributes across cellular membranes and preferentially accumulates in mitochondria in a membrane potential–dependent manner. Changes in DiOC_6_ fluorescence intensity reflect alterations in mitochondrial membrane potential and can therefore be used to monitor mitochondrial polarization in live cells (Petit et al. 1996). Because DiOC_6_ can also label other intracellular membranes, including the endoplasmic reticulum, its use requires careful optimization of dye concentration and appropriate experimental controls (Koning et al. 1993).

#### 2.1.4 Redox State

Mitochondria are major intracellular sources of reactive oxygen species (ROS). These molecules are primarily generated by electron leakage from the ETC, leading to the partial reduction of oxygen and the formation of superoxide, which is subsequently converted into hydrogen peroxide (H_2_O_2_). H_2_O_2_ can diffuse to other cellular compartments and acts both as a signaling molecule and as a potential source of damage. Peroxides are efficiently scavenged by two main enzymatic systems: heme‐based catalases and thiol‐based peroxidases, including peroxiredoxins and glutathione peroxidases.

To study intracellular H_2_O_2_ levels, a range of genetically encoded redox biosensors has been developed. Among the most widely used are the roGFP2 derivatives fused to peroxiredoxins and the HyPer family; the original HyPer derives from the bacterial redox‐sensitive regulatory transcription factor OxyR of *Escherichia coli* (Pedre 2024; Belousov et al. 2006). Both reporter families exhibit ratiometric fluorescence changes in response to oxidation, allowing the measurement of steady‐state H_2_O_2_ levels as well as the cellular scavenging capacity. These fluorescence changes can be monitored by fluorescence microscopy, plate readers, or flow cytometry. Episomal plasmids allowing the expression of two sensitive H_2_O_2_ biosensors, roGFP2‐Tpx1.C169S and HyPer7, in the cytosol of fission yeast have been successfully used (Carmona et al. 2019; de Cubas et al. 2021).

Only members of the HyPer family have been successfully targeted to different subcellular compartments. For mitochondria, HyPer7 has been fused to a mitochondrial targeting sequence (MTS), such as that of Aconitase 1, to direct the sensor to the mitochondrial matrix. Using this MTS‐HyPer7 expressed from an episomal plasmid, it has been shown that steady‐state H_2_O_2_ levels in the matrix are in the submicromolar range (approximately 0.15 µM) and that a peroxide gradient of 200:1 exists between the mitochondrial matrix and the cytosol (de Cubas et al. 2021). This sensor has been widely used to investigate redox dynamics in different genetic backgrounds, pharmacological treatments, and environmental conditions.

### 2.2 In Vitro Experimental Approaches to Monitor Metabolism

Mitochondrial function can also be studied using mitochondria‐enriched fractions, which complement and validate observations obtained from in vivo studies. Mitochondrial enrichment involves the preparation of subcellular fractions containing a high proportion of intact and functional mitochondria. In fission yeast, this procedure typically begins with enzymatic removal of the cell wall to generate protoplasts, commonly using lytic enzymes such as zymolyase in an osmotically stabilized buffer (e.g., sorbitol) to prevent cell lysis. Protoplasts are then gently disrupted using a glass homogenizer, allowing controlled breakage of the plasma membrane while preserving organelle integrity.

From this point, two main strategies are commonly employed. One approach relies on density gradient centrifugation, typically using sucrose gradients, to separate mitochondria based on their buoyant density (Meisinger et al. 2000). Alternatively, differential centrifugation protocols use sequential centrifugation steps at increasing speeds to progressively enrich mitochondrial fractions. Both methods exploit the distinct size and density of mitochondria relative to other cellular components (Chiron et al. 2007).

Mitochondria‐enriched preparations retain substantial metabolic activity and are suitable for a wide range of downstream applications. We include in this section the description of in vitro techniques to study ETC composition by Blue Native polyacrylamide gel electrophoresis (BN‐PAGE) (Section 1.2.1), oxygen consumption in mitochondrial‐enriched samples (Section 1.2.2), or enzymatic assays to measure complex IV activity (Section 1.2.3). A schematic view of these approaches is included in Figure 1B.

#### 2.2.1 BN‐PAGE to Study ETC Composition and Supercomplex (SC) Formation

BN‐PAGE is a powerful technique to analyze the composition, organization, and assembly of mitochondrial ETC complexes and SCs. This method preserves native protein–protein interactions, allowing the separation of individual complexes as well as higher‐order assemblies, named SCs. In fission yeast, SCs include the association of a dimer of complex III with one or two complex IV (Schägger 2001).

Mitochondria‐enriched fractions are solubilized with mild non‐ionic detergents, such as digitonin or dodecyl‐β‐D‐maltopyranoside (DDM). Digitonin is a non‐ionic detergent that maintains the integrity of SCs. In contrast, DDM maintains the integrity of individual complexes but not the SCs. Samples are then loaded onto native gradient polyacrylamide gels (3%–12% acrylamide) that run in a Coomassie staining buffer that serves as a protein loading control. After electrophoresis, individual complexes can be visualized using in‐gel activity assays or by immunoblotting with complex‐specific antibodies (Herbert et al. 2021; Luo et al. 2024; Timon‐Gomez et al. 2020).

BN‐PAGE allows the assessment of ETC composition under different genetic backgrounds, growth conditions, or pharmacological treatments. It can reveal changes in SC assembly, the stoichiometry of individual complexes, and alterations in complex stability that are not detectable by standard denaturing SDS‐PAGE (Herbert et al. 2021). This approach is particularly useful for linking the structural organization of the respiratory chain with functional outcomes, such as oxygen consumption or ATP production.

#### 2.2.2 Oxygen Consumption in Enriched Mitochondrial Samples

Mitochondria‐enriched fractions can be used to measure oxygen consumption in vitro using Clark‐type oxygen electrodes, as described above. In this setup, mitochondria are suspended in a respiration buffer that preserves organelle integrity, typically containing osmotic stabilizers such as sorbitol.

Respiration is initiated by the addition of appropriate substrates and cofactors that feed electrons into the ETC, together with ADP, to stimulate oxidative phosphorylation. Depending on the experimental design, substrates may include NADH‐linked sources (e.g., malate and pyruvate) or other respiratory substrates (Moore et al. 1992). Under these conditions, isolated mitochondria support electron transport and oxygen consumption in a controlled environment and remain responsive to pharmacological modulators. Inhibitors such as antimycin A block electron flow at complex III, whereas uncouplers such as FCCP dissipate the proton gradient, allowing maximal electron transport independently of ATP synthesis.

Measurements of oxygen consumption should be interpreted in conjunction with assessments of mitochondrial integrity and content. Enzymatic markers such as citrate synthase activity are commonly used as indicators of mitochondrial mass and quality, ensuring that differences in respiratory rates reflect functional changes rather than variations in mitochondrial recovery or damage during isolation (Yarian et al. 2006).

#### 2.2.3 Complex IV Activity

The activity of individual respiratory chain complexes can be assessed using mitochondria‐enriched fractions. Complex IV (cytochrome c oxidase) activity can be measured by two complementary approaches: spectrophotometric assays and in‐gel activity staining.

For spectrophotometric measurements, complex IV activity is determined by monitoring the oxidation of reduced cytochrome c at 550 nm. The decrease in absorbance reflects electron transfer from cytochrome c to molecular oxygen catalyzed by complex IV (Lemaire and Dujardin 2008). Enzymatic activity is typically normalized to citrate synthase activity, which serves as a marker of mitochondrial content (Yarian et al. 2006).

Alternatively, complex IV activity can be analyzed by in‐gel activity assays following BN‐PAGE, as previously described. After electrophoresis, gels are incubated in a reaction buffer containing reduced cytochrome c as substrate and 3,3′‐diaminobenzidine (DAB). Cytochrome c oxidation by complex IV leads to DAB oxidation and polymerization, forming a brown precipitate at sites of enzymatic activity (Timon‐Gomez et al. 2020). Signal intensity can be normalized to total protein loading.

A key advantage of the in‐gel assay is that it enables discrimination between complex IV activity within SC assemblies and that of the individual complex, providing structural and functional information simultaneously.

## 3 Mitochondrial Homeostasis

To ensure the proper execution of mitochondrial functions, mitochondrial homeostasis must be tightly regulated. Mitochondria are double‐membrane organelles composed of an outer and an inner membrane, which delimit the intermembrane space and enclose the mitochondrial matrix. Beyond this structural organization, mitochondria are highly dynamic organelles that undergo continuous structural remodeling to adapt to changing environmental and metabolic conditions.

From a morphological perspective, mitochondria in yeast typically form an interconnected tubular network that extends throughout the cell, facilitating the efficient distribution of energy and metabolites. Mitochondrial morphology is regulated through coordinated changes in mitochondrial number and through the balance between fusion and fission events (Lackner 2014; van der Bliek et al. 2013). The equilibrium between these two opposing processes is essential to maintain a tubular mitochondrial network and overall mitochondrial functionality (Gomes et al. 2011).

Mitochondrial fusion involves the merging of both the outer and inner mitochondrial membranes and is mediated by conserved mitofusins. In fission yeast, mitochondrial outer membrane fusion is primarily regulated by Fzo1, a conserved mitofusin‐like GTPase (Rapaport et al. 1998), while inner membrane fusion requires the dynamin GTPase Msp1 (Leroy et al. 2010). Mitochondrial fusion promotes respiratory metabolism by enabling the homogeneous distribution of mitochondrial enzymes, metabolites, and mitochondrial DNA, as well as by facilitating the equalization of ROS within the mitochondrial network (Tábara et al. 2025). As determined by us and others (Dong et al. 2022; Vega et al. 2022), Δ*msp1* and Δ*fzo1* cells display polarized fragmented mitochondria due to their inability to promote mitochondrial fusion.

Conversely, mitochondrial fission is an equally essential process that enables mitochondrial segregation and quality control. In *S. pombe*, mitochondrial fission is mediated by the dynamin‐related GTPase Dnm1 and its adaptor proteins, including Fis1, an outer mitochondrial membrane protein that recruits the fission machinery (Mozdy et al. 2000; Otsuga et al. 1998). Mitochondrial fission plays a key role in isolating damaged or dysfunctional mitochondrial fragments, which can then be selectively removed through mitophagy (Gomes and Scorrano 2013). As described using fluorescence microscopy (Vega et al. 2022), the mitochondrial morphology of Δ*fis1* and Δ*dnm1* reveals fission defects and displays networks of hyperfused mitochondria.

In fission yeast, mitophagy is a selective process that relies on the core autophagy machinery but requires mitochondria‐specific receptors. One such receptor is Atg43, which localizes to the outer mitochondrial membrane and mediates the recognition and targeting of mitochondria for autophagic degradation (Fukuda et al. 2020; Fukuda and Kanki 2021). Proper mitochondrial turnover through mitophagy is essential to maintain a healthy mitochondrial population and is considered a hallmark of healthy aging (Vega et al. 2022).

In addition to changes in overall mitochondrial morphology, mitochondrial homeostasis also involves the remodeling of mitochondrial cristae. Cristae are invaginations of the inner mitochondrial membrane that serve as platforms for the assembly of oxidative phosphorylation complexes (Cogliati et al. 2013). Cristae architecture can vary from loosely organized to highly compacted structures depending on respiratory demand. Under conditions of high energy requirement, respiratory complexes reorganize into higher‐order assemblies or SCs, already described above. In *S. pombe*, two main SCs have been described, consisting of a dimer of complex III associated with one or two copies of complex IV (Schägger 2001). The cryo‐electron microscopy structure of complex IV in fission yeast has recently been reported (Moe et al. 2023). The formation of these SCs is thought to enhance electron transfer efficiency, minimize electron leakage and ROS production, and optimize mitochondrial respiration (Bianchi et al. 2004).

The main experimental approaches used to assess mitochondrial morphology (Section 2.1) and mitophagy (Section 2.2) are described below, and a schematic view of these approaches is included in Figure 1C.

### 3.1 Experimental Approaches to Monitor Morphology

The regulation of mitochondrial morphology, dynamics, and turnover is essential for maintaining mitochondrial function and overall cellular homeostasis. As an example of that, defects in mitochondrial fusion lead to a highly fragmented mitochondrial network, a phenotype that has been shown to severely impair mitochondrial respiration. Cells harboring fragmented mitochondria are unable to grow on non‐fermentable carbon sources, lose mitochondrial membrane potential due to defective respiratory activity, and display impaired mitochondrial protein import (Dong et al. 2022). These defects compromise multiple mitochondrial functions and ultimately result in reduced chronological lifespan. For these reasons, accurately assessing mitochondrial homeostasis is critical for understanding mitochondrial function.

#### 3.1.1 Fluorescent Labeling of Mitochondrial Proteins

Fluorescent tagging of mitochondrial proteins with fluorescent tags such as GFP or mCherry is a widely used approach to visualize mitochondrial morphology in living cells. A critical consideration when selecting a target protein for tagging is ensuring that the fusion protein does not lose function and compromise mitochondrial integrity. Certain tags can perturb mitochondrial activity, and such defects are often readily detectable through alterations in mitochondrial morphology. Tagging strategies that impair mitochondrial function typically result in the loss of the tubular mitochondrial network and an increased proportion of fragmented mitochondria, frequently accompanied by reduced respiratory capacity compared with wild‐type cells.

One tagging strategy that has been shown to preserve mitochondrial function is the fluorescent labeling of the succinate dehydrogenase subunit Sdh2 with GFP or mCherry. In general, mitochondria can be classified into three main morphological states: tubular (wild‐type–like), fragmented, or hyperfused, with additional variations of these core morphologies also observed. As an example, fragmented mitochondria can adopt a polarized configuration or be distributed throughout the entire cell. When analyzing mitochondrial morphology in a given genetic background or experimental condition, it is essential to include appropriate reference controls representing these three morphological states. Wild‐type cells provide a reference for the tubular mitochondrial network. As controls for hyperfused mitochondria, fission‐deficient mutants such as Δ*fis1* or Δ*dnm1* are commonly used, as these strains display extended and highly interconnected mitochondrial networks throughout the cell. Conversely, defects in mitochondrial fusion, such as those resulting from disruption or tagging of *msp1*, lead to highly fragmented mitochondrial structures and serve as suitable controls for this morphological state (Vega et al. 2022). The inclusion of these well‐defined reference strains enables robust and comparative assessment of mitochondrial morphology across different experimental contexts.

#### 3.1.2 Membrane Potential–Independent Mitochondrial Dyes

As an alternative to genetically encoded fluorescent tags, mitochondrial morphology can also be visualized using dyes that accumulate in mitochondria independently of the mitochondrial membrane potential. One such dye is MitoTracker Green FM, a fluorescent probe that labels mitochondria regardless of their energetic state. MitoTracker Green FM associates with mitochondrial membranes and allows the visualization of mitochondrial morphology even under conditions in which membrane potential is compromised, such as in respiratory‐deficient mutants or upon pharmacological inhibition of the respiratory chain. This property makes it particularly useful for assessing mitochondrial structure independently of mitochondrial activity. As with other fluorescent probes, careful optimization of dye concentration and incubation conditions is required to minimize nonspecific staining and phototoxic effects.

### 3.2 Experimental Approaches to Monitor Mitophagy

Mitophagy is a selective process responsible for the degradation of damaged or dysfunctional mitochondria and depends on the core autophagy machinery. In this process, the mitophagy receptor Atg43 localizes to the outer mitochondrial membrane, where it interacts with components of the autophagosome formation machinery, promoting the engulfment of mitochondria destined for degradation within the vacuole. Several conditions have been shown to induce mitophagy in yeast, including nitrogen starvation and cellular aging (Kohda et al. 2007; Vega et al. 2022; Zhao et al. 2016). In particular, cells grown under respiratory conditions, such as low‐glucose media, exhibit increased mitophagy, reflecting their higher reliance on mitochondrial respiration and the associated demand for mitochondrial turnover to sustain a healthy mitochondrial pool (Vega et al. 2022).

#### 3.2.1 Immunoblot

Mitophagy activation is commonly monitored by immunoblot analysis through the detection of cleavage of mitochondria‐targeted fluorescent fusion proteins. One widely used reporter is the already mentioned succinate dehydrogenase subunit Sdh2 tagged with GFP or mCherry. It is essential that the fusion protein does not alter mitochondrial function or morphology, as such perturbations can indirectly affect mitophagy. Under basal conditions, immunoblotting detects the full‐length fusion protein. Upon induction of mitophagy, mitochondria are delivered to the vacuole, where most mitochondrial proteins are degraded; however, GFP is relatively resistant to vacuolar proteolysis due to its stable folding, resulting in the accumulation of free GFP. The appearance of a free GFP band therefore serves as a biochemical readout of mitophagy. As positive controls for mitophagy induction, cells can be subjected to nitrogen starvation for approximately 16 h or cultured for two to 3 days to reach stationary phase in low‐glucose medium (e.g., YE5S containing 1% glucose), conditions under which the accumulation of free GFP can be readily detected by immunoblot (Kohda et al. 2007; Vega et al. 2022; Zhao et al. 2016).

#### 3.2.2 Fluorescence Microscopy

An alternative approach to study mitophagy relies on fluorescence microscopy–based techniques. This strategy aims to assess the colocalization of mitochondria with vacuolar markers as a readout of mitochondrial delivery to the vacuole. For this purpose, mitochondria can be labeled by tagging a mitochondrial protein such as the succinate dehydrogenase subunit Sdh2 with GFP or mCherry, while the vacuole is marked using a vacuolar protein labeled with a spectrally distinct fluorophore. A commonly used vacuolar marker is carboxypeptidase Y (Cpy1), a resident vacuolar hydrolase (Tabuchi et al. 1997). Under conditions that induce mitophagy, mitochondrial fluorescence is expected to colocalize with the vacuolar signal, indicating the sequestration of mitochondria within the vacuole (Zhao et al. 2016).

As with immunoblot‐based assays, appropriate positive controls are essential to validate mitophagy induction. These include cells subjected to nitrogen starvation for at least 16 h (Kohda et al. 2007), as well as chronologically aged cells grown under respiratory conditions, such as low‐glucose media (YE5S 1% glucose), typically analyzed after 3 days of growth (Vega et al. 2022). These conditions robustly induce mitophagy and facilitate the interpretation of microscopy‐based colocalization assays.

## 4 Conclusions

Over the last years, *S. pombe* has emerged as a powerful and versatile model to study mitochondrial efficiency. Its obligate dependence on mitochondrial respiration, even under fermentative conditions, makes fission yeast particularly well suited to dissect mechanisms that closely resemble those operating in mammalian cells. Some future directions which are currently underexplored in fission yeast include the study of mitochondrial protein import (Vega et al. 2022), the analysis of mitochondrial protein synthesis (Bykov and Schuldiner 2024), the role of mitochondria in cytosolic protein quality control (Schlagowski et al. 2021), mitoproteome quantitative composition (Di Bartolomeo et al. 2020) or the in‐depth study of mitochondrial protein partners (Gouget et al. 2008). The experimental strategies discussed in this review enable the integrated analysis of mitochondrial function by linking respiratory activity, energetic state, redox balance, and organelle architecture in living cells and isolated mitochondria. Together, these approaches establish *S. pombe* as an ideal model to explore how mitochondrial organization and behavior shape cellular metabolism and adaptive responses.

## Conflicts of Interest

The authors declare no conflicts of interest.

## Data Availability

Data sharing not applicable to this article as no data sets were generated or analysed during the current study.
